# Orthorexia and anorexia nervosa: two distinct phenomena? A cross-cultural comparison of orthorexic behaviours in clinical and non-clinical samples

**DOI:** 10.1186/s12888-017-1241-2

**Published:** 2017-02-21

**Authors:** C. Gramaglia, A. Brytek-Matera, R. Rogoza, P. Zeppegno

**Affiliations:** 1 Psychiatry Institute, Dipartimento di Medicina Traslazionale, Università del Piemonte Orientale, Via Solaroli n° 17, 28100 Novara, Italy; 2 SWPS University of Social Sciences and Humanities, Katowice Faculty of Psychology, Katowice, Poland; 3 University of Cardinal Stefan Wyszyński, Warsaw, Poland

**Keywords:** Anorexia nervosa, Orthorexia, Culture, ORTO-15

## Abstract

**Background:**

Orthorexia nervosa (ON) is defined as pathological healthful eating. The aim of this study was to investigate whether there is any difference in orthorexic behaviours between clinical and non-clinical groups, and in different cultural contexts. .

**Methods:**

Recruitment involved both female patients with anorexia nervosa (AN) and healthy controls (HC) from Italy and Poland (*N* = 23 and *N* = 35 AN patients; and *N* = 39 and *N* = 39 HCs, in Italy and Poland, respectively). Assessment of orthorexic behaviours was performed with the ORTO-15 test.

**Results:**

Statistically significant differences were found between Italian women in the AN and HC group, whereas no difference between Polish women in the AN and HC group was found. Both Italian groups scored significantly higher than the Polish ones on the ORTO-15.

**Conclusions:**

Differences have been found between the Italian and Polish samples, both in the percentage of individuals with orthorexic behaviours as suggested by an ORTO 15 score below the cutoff, and in the mean ORTO 15 scores in the AN and HC groups, suggesting cross-cultural differences in orthorexic behaviours, whose meaning is currently difficult to understand.

## Background

Orthorexia nervosa (ON) is defined as an exaggerated, obsessive, pathological fixation on healthy food, eating healthy, or rather health-conscious eating behaviours [1–3]. The literature is not unequivocal about whether ON is an eating disorder (at all), a variant of a currently recognized eating disorder (ED), such as the avoidant/restrictive food intake disorder, or a different disorder [4–6]. Similarities and possible overlaps have been suggested with the spectrum of obsessive-compulsive, eating, or somatoform disorders [5, 7, 8]. Orthorexia shares issues with disturbed eating/nutrition attitudes as shown by excessive focus on food-related topics [9, 10], strict diet [11], perfectionism [12], co-occurrence of anxiety, need of control [13], stiffness of behaviour and rituals related to preparation of meals [14, 15]. In individuals with ON, the most characteristic features of anorexia nervosa (AN) and bulimia nervosa (BN) are absent, namely excessive preoccupation with losing weight, extreme fear of gaining weight and body size overestimation [12, 16]. Reports from the literature are far from being consistent, nonetheless a high frequency of occurrence of orthorexic behaviours in patients with EDs has been described, and eating related disturbances may play the role of risk factors for orthorexia, although the reverse is also possible [17–23]. Overall, despite increasing research efforts, there is still a dearth of empirical data regarding the relation between AN and orthorexia.

Reports are not unequivocal also as far as the correlation between eating pathology (as measured for instance with the Eating Attitudes Test) and orthorexia (as measured with the ORTO-15 test) is concerned [17, 24–28]. Moreover, while orthorexia entails both a fixation, i.e. a mental process, and specific behaviours, the ORTO-15 test, especially when using a score of 40 as threshold value, likely measures the latter (healthy eating behaviours) rather than the first (fixations and obsessive traits) [3, 29].

The aim of the present research was to assess the possible overlap between orthorexia, measured with the ORTO-15 test, and AN. For this purpose, we assessed a sample of female patients under treatment for a diagnosis of AN, and a sample of female healthy controls (HCs) from the general population. The second aim was to assess the possible cross-cultural differences in orthorexic behaviours between Italian and Polish women, both with and without a diagnosis of AN. For this purpose, the same research procedure was carried on in two culturally different countries – Italy and Poland.

## Methods

Patients with a diagnosis of AN according to DSM-5 criteria [30] were recruited both in Italy (Psychiatry Institute, Università del Piemonte Orientale, AOU Maggiore della Carità, Novara), and in Poland (the Polish National Center for Eating Disorders, Wroclaw) (*N* = 23 and *N* = 35, respectively), as well as HCs from the general population (*N* = 39, both in Italy and Poland). All groups included individuals aged > 18 years, who voluntarily took part in the study after informed consent was obtained. The need for approval from the local ethics committee was waived since the study did not entail anything beyond standard and everyday clinical practice.

The ORTO-15 test [3, 29] was used as a measure of orthorexic behaviours. The ORTO-15 is composed of 15 items with closed multiple-choice answers (“always”, “often”, “sometimes”, “never”). Items investigate the obsessive attitude of the individuals in choosing, buying, preparing and consuming food they consider to be healthy. A score equal to 1 for each item corresponds to an orthorexic tendency in the eating behaviour, while a score equal to 4 points indicates normal eating habits. Higher scores suggest normal eating habits; a cutoff of 40 or 35 points has been suggested. We used the 40-points cutoff, which according to Donini and coworkers [29] allows the identification of symptoms consistent with orthorexia nervosa with a sensitivity of 100.0%, a specificity value of 73.6%, a positive predictive value of 17.6% and a negative predictive value of 100%. In our study we used both the Italian and Polish [31] validated versions of the questionnaire.

Statistical analyses were performed using Chi-square test and parametric t tests for independent sample to test the study hypotheses; statistical significance level was set at p-value <0.05. All analyses were performed with SPSS v.22 [32].

## Results

Mean age and BMI of AN and HCs from both countries are reported in Table 1, as well as the percentage of individuals scoring under the ORTO-15 cutoff (i.e. with orthorexic tendencies). No difference was found with the Chi-square test in the percentage of subjects scoring under the ORTO-15 cutoff when comparing Italian AN and Italian HC (*p* = .263), or when comparing Polish AN and Polish HC (*p* = .670). On the contrary, the percentage of individuals with orthorexic behaviours as described by an ORTO-15 score below the cutoff was different between Italian AN and Polish AN (*p* = .031) and between Italian and Polish HC (*p* = .001). Descriptive statistics and normality distribution test of ORTO-15 scores in the AN and HC groups are presented in Table 2. Data in all studied samples as divided by country were normally distributed, except for the Italian AN group, likely due to the small number of patients included. Nonetheless, since both kurtosis and skewness in all samples did not exceed 1, parametric statistics were used for the analyses.

**Table 1:** Mean age, mean BMI, and % of individuals scoring under the ORTO-15 40-points cutoff

Sample	Mean age (years)	Mean BMI (Kg/m^2^)	% scoring under the ORTO-15 40-points cutoff
Italian AN	30.39	16.36	60.9%
Italian HC	34.41	22.69	46%
Polish AN	22.97	17.21	85.6%
Polish HC	23.00	21.69	82%

**Table 2:** Descriptive statistics and normality distribution test of ORTO-15 scores in the AN and HC groups (bold is for statistically significant results)

Sample	*N*	*M*	*SD*	Kurtosis	Skewness	Kolmogorov-Smirnov normality test	*p*
Italian AN	23	37.21	1.15	–0.97	–0.07	0.20	**.024**
Italian HC	39	39.41	0.50	0.61	0.31	0.12	.191
Polish AN	35	34.37	0.83	–0.88	–0.20	0.13	.166
Polish HC	39	35.36	0.58	–0.74	–0.21	0.10	.200

The results of the *t*-tests are presented in Table 3. First, groups recruited in the same nation were compared. The statistical difference between Italian AN and HC groups was found at the boundary of the accepted threshold, whereas no differences between Polish women in the AN and HC group was found. Second, women in the AN and HC groups were compared, independently. The Italian groups scored significantly higher than the Polish ones, both for AN and HC group.

**Table 3:** Comparison of the mean ORTO-15 score in AN and HC groups divided by country (bold is for statistically significant results)

Group 1	Group 2	*t*_(df)_	*p*
Italian AN	Italian HC	–2.00_(60)_	**.049**
Polish AN	Polish HC	–0.98_(72)_	.325
Polish AN	Italian AN	–2.06_(56)_	**.044**
Polish HC	Italian HC	5.31_(76)_	**.001**

## Discussion

Italian patients were older than Polish ones; regrettably data about illness duration were not available, although it is likely that the Italian AN group included patients with a more severe and enduring ED, as suggested also by the lower BMI values.

In the current Italian HC sample, 54% scored 40 or above at the ORTO-15, suggesting the presence of orthorexic behaviours in 46% of the sample, consistent with a previous study performed in Italy and reporting a prevalence of orthorexia nervosa of 57.6% (40-point threshold, ORTO-15), or 21% (35-point threshold, ORTO-15) [19].

Surprisingly, in the Polish HC sample the percentage of those scoring 40 or above (i.e. those with no orthorexic tendencies) was only 18%, with 82% of the sample self-reporting orthorexic behaviours as described by the ORTO-15 test. Mean scores under the 40-points cutoff [33], and percentages up to 68.55% of orthorexic behaviours measured with the ORTO-15 [34] have been described in Poland.

Differences in the mean score at the ORTO-15 were found between AN and HC groups in Italy, but not in Poland. Whether there is an overlap or not between AN and orthorexic behaviours, and whether this is also mediated by cultural factors should be further assessed by studies involving larger samples.

The Chi-square differences in the percentage of orthorexic behaviours suggested by an ORTO-15 score below the cutoff, and the finding of higher ORTO-15 mean scores in Italian women than in Polish ones, independent of belonging to either the AN or the HC group, suggest cross-cultural differences in orthorexic attitudes and behaviours. Their meaning is currently difficult to understand, considering the lack of data about this issue. These differences are likely due to culture-related discrepancies in the approach to food and health concept. Italy has a widely-acknowledged culture about eating and nutrition, and the Mediterranean diet has long been praised for its benefits for overall health [35, 36], but has a strong focus on taste as well. In Poland, in the last ten years, good consumer practices have gained a slightly greater popularity (e.g. checking the composition of foodstuffs). For Poles, healthy eating is the most important among various activities aimed at improving their own state of health; eight out of ten adults report eating healthy, and most Poles assess their diet as balanced [37]. This may explain the high percentage of people scoring under the ORTO-15 cutoff, i.e. self-reporting orthorexic behaviours.

Further studies are warranted to assess whether healthism or Alternative Food Networks (AFN) play a role in the differences between Italian and Poles. Healthism is a concept proposed to discuss and contextualize orthorexia, which would be an advocate for healthy eating to pursue a better health [38]. Orthorexic tendencies have been described in people actively engaged in AFN [39], but while most people are likely to do so for the benefits that this would bring to society, health and environment, currently it cannot be excluded that a small number of individuals may rather try to disguise disordered eating attitudes behind these socially acceptable (and even laudable) habits.

Limitations of this study are the relatively small sample size, which may hinder the generalizability of the current results, and the intrinsic flaws of the ORTO-15. As far as the former is concerned, regrettably it is a shared problem of most studies in the field of orthorexia [18]. As for the latter, recently it has been claimed that new tools are necessary for the assessment of orthorexia, in consideration of the limits of both the ORTO-15 and Bratman’s Test [8, 40–42], which tend to overestimate its prevalence. While the ORTO-15 is still the most used instrument by the studies in this field, we cannot exclude the possibility that other assessment tools would yield different results. Moreover, the ORTO-15 alone is not a diagnostic tool, and regrettably in this study neither a measure of personality features was available [3] nor the suggested diagnostic criteria for ON [2, 43] were used.

Last, since this was a naturalistic study based on voluntary participation, the samples in Poland and Italy were not age matched. Therefore, we cannot exclude that some of the differences we found depend on age issues.

## Conclusions

In the current sample, orthorexic behaviours did not overlap with AN in the Italian samples, and seemed to be influenced by cultural issues. Further studies are warranted to assess whether orthorexia is just a variant of individuals’ ways of approaching eating and nutrition, or something different, and to disentangle the meaning of cultural differences in its prevalence.

## Abbreviations

AFN: Alternative Food Networks
AN: Anorexia nervosa
BN: Bulimia nervosa
ED: Eating disorder
HCs/HC: Healthy controls
ON: Orthorexia nervosa
